# Genetic Landscape of Familial Melanoma

**DOI:** 10.3390/genes16080857

**Published:** 2025-07-23

**Authors:** Carmela Scarano, Iolanda Veneruso, Valeria D’Argenio

**Affiliations:** 1 CEINGE-Biotecnologie Avanzate Franco Salvatore, 80145 Napoli, Italy; scaranoc@ceinge.unina.it (C.S.); dargenio@ceinge.unina.it (V.D.); 2 Department of Molecular Medicine and Medical Biotechnologies, Federico II University, 80131 Napoli, Italy; 3 Department for the Promotion of Human Sciences and Quality of Life, San Raffaele Roma Open University, 00166 Rome, Italy

**Keywords:** familial melanoma, germline mutations, genes panel testing, familial cancer, hereditary cancer syndrome, molecular diagnosis

## Abstract

About 10% of all forms of melanoma occur in a familial context and may be due to germline predisposing mutations transmitted as autosomal dominant traits within the affected families. *CDKN2A* is a highly penetrant gene associated to familial melanomas, being responsible of up to 40% of the cases. Other high, moderate, and low penetrance genes are being discovered, even if their own contribution to melanoma risk is still under debate. Indeed, next generation sequencing-based strategies enable large genomic regions to be analyzed, thus identifying novel candidate genes. These strategies, in diagnostic settings, may also improve the identification of the hereditary cases between all melanomas. The identification of the at-risk subjects gives an important opportunity for cancer surveillance in order to reduce the risk of onset and/or make early diagnosis. In addition, the identification of molecular biomarkers may drive the future development of specific targeted therapies, as already done for other inherited cancer syndromes. Here, we summarize the state of the art regarding the molecular basis of the hereditary susceptibility to develop melanoma.

## 1. Introduction

Melanoma is the most lethal skin cancer and its incidence has risen over the last decades, currently being the 17th most common cancer in both sexes. According to GLOBOCAN 2020 (https://gco.iarc.fr/, accessed on 20 July 2025) and the National Cancer Institute (https://seer.cancer.gov/statfacts/html/melan.html, accessed on 20 July 2025), the global incidence of melanoma is estimated at 21.9 cases per 100,000 persons per year, with the highest rates reported in Australia and New Zealand (up to 55–60/100,000). The incidence has continued to rise in recent decades, particularly among fair-skinned populations in Western countries. About 300,000 new cases have been diagnosed worldwide just in 2022 (https://www.wcrf.org/, accessed on 20 July 2025) and it has been estimated that in 2025 about 104,960 new melanomas will be diagnosed and about 8430 people will die of melanoma, only in the US (https://www.cancer.org/, accessed on 20 July 2025). Melanomas may develop through a multi-step process that start from benign, pre-cancerous lesions (nevus); however, most of them are developed de novo and it has also been reported that melanomas arising on a nevus sometimes do not share driver mutations with the preexisting nevus, suggesting that they may be two different and independent entities [1,2,3,4,5,6]. Even if most melanomas are sporadic and due principally to environmental risk factors, about 10% of all melanoma cases are familial and may be due to germline mutations in predisposing genes [7,8]. To date, several genes have been identified as risk factors for familial melanoma and, based on the probability of developing a form of melanoma if mutated, they are commonly classified into high, medium, and low penetrance genes [7,8]. As with other inherited cancers, recognizing individuals carrying an inherited predisposition to develop melanoma has important implications for identifying at-risk subjects within affected families and implementing preventive programs aimed at reducing cancer onset or enabling earlier diagnosis. Recent technological advances in the genomics field have made it possible to study multiple genomic loci, in several patients with a high resolution and at progressively reducing costs [9,10]. Accordingly, multiple genes panel testing is becoming commonly offered for the molecular diagnosis of an increasing number of human diseases, including hereditary cancers and also melanoma [11,12,13]. In this review, we will describe the different high, medium, and low penetrance genes that have been associated to date to a hereditary risk of melanoma, the diagnostic tests, and the recommendations currently available for the identification and management of the mutations’ carriers.

## 2. Methods

A comprehensive literature search was conducted using PubMed and the following keywords: “familial melanoma”, “familial melanoma predisposition”, “familial melanoma germline mutations”, “familial melanoma predisposing genes”, “*CDKN2A* germline variants”, “familial melanoma high penetrant genes”, “familial melanoma moderate penetrant genes”, “familial melanoma low penetrant genes”, “novel familial melanoma genes”, “familial melanoma susceptibility loci”, and “familial melanoma management”. As temporal windows, publications from 2000 to 2025 were searched; however, a manual search for the oldest references mentioned in the found articles was also carried out. Inclusion criteria required peer-reviewed original articles, meta-analyses, reviews, and guideline-based recommendations. Non-English reports were excluded. Papers in the search results reporting somatic mutations were not included since they are out of the topic of the present review. Clinvar and dbSNP databases have been used for variants assessment.

## 3. Defining Familial Melanoma Concept

Melanomas can develop in a familial context suggesting that a genetic susceptibility to melanoma development may be inherited within these affected families. Familial melanoma (7–15% of all melanomas) is defined as the occurrence of three or more melanoma cases within the same family branch, or at least two cases among first-degree relatives. Within these are defined as “hereditary melanomas”, those cases related to the presence of a germline pathogenic/likely pathogenic variant in a melanoma susceptibility gene (2% of all melanomas and about 20% of familial melanomas) [14].

Thus, in the presence of a personal history of multiple primary melanomas, and/or melanomas diagnosed in at least two first-degree relatives, and/or in families with at least three melanoma cases, genetic testing and counseling should be carried out to assess the presence of germline predisposing mutations in the proband and, consequently, in his/her family. It is important to underline that the criteria to include melanoma patients into genetic counseling may vary between countries taking into account the highest incidence in some geographic areas (i.e., USA and Australia) and also patient’s features, like the age at diagnosis [15,16,17,18,19].

The cyclin-dependent kinase inhibitor 2A (*CDKN2A*) gene is the most commonly mutated gene in familial melanoma; however, *CDKN2A* mutations explain up to 40% of hereditary cases [20,21]. Even if other susceptibility genes are being discovered (and other are probably yet to be discovered), it has also been suggested that a polygenic inheritance, featured by the co-occurrence of multiple low-risk variants and phenotype-modifier genes, may be involved. All the genes that have been associated to an increased risk of melanoma are reported in the next paragraphs according to their penetrance (Table 1).

Genes related to other hereditary cancer syndromes in which melanoma may be a “secondary” cancer, such as *BRCA1/2*, *TP53,* and *PTEN*, will be not covered by the present review; please refer to specific papers on these topics for more details [22,23,24,25]. 

### 3.1. High-Risk Genes

**Figure 1:**
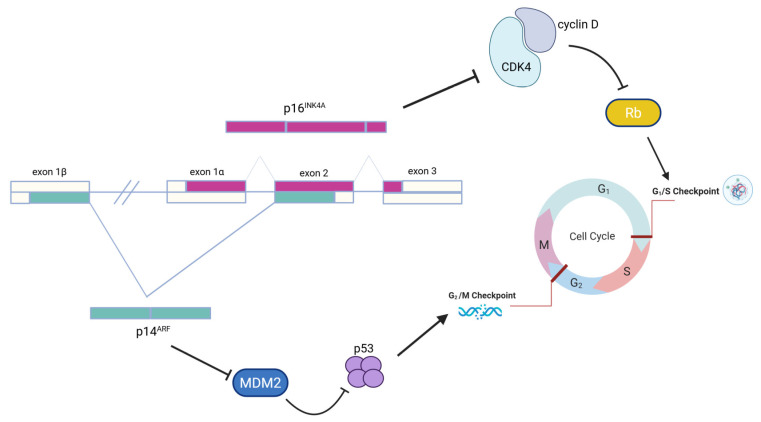
*CDKN2A* gene-encoded proteins and their role in cells cycle regulation.

As mentioned above, within the highly penetrant genes, the most commonly associated to hereditary susceptibility to melanoma is the *CDKN2A* gene [18]. Indeed, while the overall probability to find a germline *CDKN2A* mutation in people with melanoma is lower than 2%, this value raises up to 20–40% in familial melanomas [19]. The *CDKN2A* gene is located on chromosome 9p21.3 and, by using two different first exons, namely 1a and 1β, it encodes for two proteins: p16INK4A (156 amino acids) and p14ARF (132 amino acids), respectively [20]. Protein p16INK4A interacts with the Retinoblastoma protein and controls the G1 to S transition during the cell cycle. Protein p14ARF also plays an important role in the cell cycle regulation process: it induces cell cycle arrest in the G2 phase and the subsequent cell death (Figure 1). In malignant melanoma, usually, the two proteins are affected together [26].

To date, 14,786 sequence variations from dbSNP (https://www.ncbi.nlm.nih.gov/snp/, accessed on 20 July 2025) are known and, among them, 163 seem to be pathogenic/likely pathogenic for the ClinVar Database (https://www.ncbi.nlm.nih.gov/clinvar/, accessed on 20 July 2025).

Interestingly, there seems to be an association between the *CDKN2A* mutations’ frequency and the geographic area: different founder mutations have been described in Northern Europe, Iceland, UK, and European Mediterranean countries (France, Italy, and Spain) [27,28,29,30,31]. Nevertheless, also considering that penetrance varies depending on other environmental and genetic factors, *CDKN2A* mutations are usually related to early age of melanoma onset, multiple primary melanomas, and a high number of affected family members [23,32].

It has to be noticed that penetrance estimates for *CDKN2A* mutations vary widely, ranging from 30% to 70% by age 80, depending on geographic region and family selection criteria. In particular, the lifetime risk has been estimated at approximately 58% in Europe and up to 91% in Australia by age 80, according to data from the International Melanoma Genetics Consortium (https://genomel.org/, accessed on 20 July 2025). However, these evaluations may be also impaired by sample size and geographic region. As reported elsewhere [14], the Mediterranean population is often underrepresented in genetic melanoma studies, thus impairing unbiased risk estimations.

In addition to familial melanoma, *CDKN2A* germline mutations have been also associated with an elevated risk of pancreatic [33], lung, and other tobacco-related cancers [7,8,27,28,29,30,31]. These features characterize the FAMMM syndrome (OMIM 155601), which includes atypical nevi, multiple primary melanomas, and a family history of melanoma and/or pancreatic cancer [30]. Finally, *CDKN2A* mutated families have shown also an increased risk to develop other type of cancers, like breast cancer [28,29,34,35,36].

The *CDK4* (Cyclin-Dependent Kinase 4) gene is located on the chromosome 12q14.1. The encoded protein is a Ser/Thr-kinase component of the cyclin D-CDK4 (DC) complex that is implicated in the phosphorylation and inhibition of retinoblastoma (RB) members, including RB1, and regulates the cell cycle during G1 to S phase transition [37]. Totally, 4485 *CDK4* sequence variants have been reported to date, seven being classified as pathogenic/likely pathogenic and associated to cutaneous malignant melanoma onset. All these *CDK4* variants are located in exon 2; in particular, two of them affect the codon 24 (p.Arg24His and p.Arg24Cys), which is involved in the binding of p16INK4A [14]. So far, *CDK4* mutations have been reported in a limited number of families from different geographic areas (including Europe, Australia, and USA). Thus, due to their low frequency, *CDK4* mutations’ contribution to familial melanoma risk still remain difficult to be accurately assessed.

*BAP1* gene (BRCA1-Associated Protein 1) is located on chromosome 3p21.1. The protein function is to help in regulating many proteins involved in different cellular processes; indeed, the BAP1 protein controls cell growth, proliferation, and cell death mechanisms. In total, 248 out of 8420 *BAP1* variants are known to be pathogenic/likely pathogenic (https://www.ncbi.nlm.nih.gov/clinvar/, accessed on 20 July 2025) [38]. These *BAP1* gene mutations have been mainly associated to uveal melanoma and to an increasing number of cancers, including mesothelioma, meningioma, and renal cell carcinoma [39]. The different phenotypic expression seems to be related to different *BAP1* gene mutations, showing variable penetrance for different tumor types [40]. However, since most of the families carrying a *BAP1* germline mutation present at least one melanoma case, the high penetrance for familial melanoma seems to be confirmed [7]. To improve the understanding regarding the spectrum of *BAP1*-related cancers, a comprehensive study on the clinical features associated to all known *BAP1* mutations has been carried out, highlighting the need for a curated registry in order to improve mutation carriers’ management [41]. However, the interpretation of *BAP1* variants remains challenging, as most published studies are based on limited family cohorts or targeted sequencing in referral populations, often lacking proper control groups.

Recent studies have shown the importance in familial melanoma susceptibility, of genes that play a role in telomere maintenance, in the regulation of telomeres length, and in their protection [42,43]. Telomers are regions of repetitive nucleotide sequences at each end of a chromosome. Their principal function is to maintain the genomic stability and chromosomal integrity protecting chromosome ends from degradation, fusions, and atypical recombinations. Telomeres became smaller with age, smoking, and UV radiations. Consequently, these factors affecting telomeres maintenance processes are perfect candidates as risk factors for the onset of cancers [26,42,43]. Four genes belonging to this class have been implicated in familial melanoma susceptibility: (1) *TERT* (Telomerase Reverse Transcriptase); (2) *POT1* (Protection of Telomeres 1); (3) *ACD* (ACD Shelterin Complex Subunit and Telomerase Recruitment Factor); and (4) *TERF2IP* (TERF2 Interacting Protein) [44,45].

The *TERT* gene is located on chromosome 5p15.33 and encodes for a catalytic subunit of telomerase involved in telomere length maintenance. A high penetrant mutation has been described so far in the *TERT* promoter region. This mutation has been found in a German family with four affected individuals, all of them carrying the same mutation [46]. A subsequent large population-based study identified this rare *TERT* promoter mutation in another melanoma family confirming its high penetrance, its association with early onset melanoma, and highlighting an increased risk of developing other cancers [47,48].

The *POT1* gene is located on chromosome 7q31.33 and encodes a protein involved in telomeres protection. Mutations affecting this gene have been reported as highly penetrant for familial melanoma occurrence [49,50]. Roblez-Espinoza et al., analyzing several families from different countries, identified *POT1* as a novel familial melanoma susceptibility gene; interestingly, they found that all *POT1* mutations carriers were affected by melanoma, and most of them had early age of onset and multiple primary cancers, suggesting the high penetrance of this gene [49]. At the same time, Shi J et al., using whole exome sequencing, identified a *POT1* founder mutation in apparently unrelated Italian families [50]. Subsequently, a novel *POT1* mutation has been reported in a family with multiple cases of primary melanomas, as well as other kind of cancers [51], suggesting that a broader range of cancers may be associated to predisposing mutations in *POT1.* A large-population study was carried out in 694 Austrian patients to assess the role of *POT1* germline mutations in predisposing familial melanoma [52]. The entire sequence analysis of *POT1* in all the study subjects identified only eight variants in high-risk melanoma patients, and only one of these has been predicted as potentially pathogenic, suggesting that *POT1* mutations are almost rare [52]. On the contrary, another study by Poltrony et al. carried out in 228 Spanish melanoma families suggests that *POT1* mutations are involved in familial melanoma susceptibility in Spain [53]. Moreover, Wong et al. described a novel *POT1* mutation supporting the need of including *POT1* analysis in the assessment of familial melanoma inherited risk [54]. Finally, Abu Shtaya et al. recently described the identification of the *POT1* p.(Ile78Thr) variant, previously classified with conflicting interpretations of pathogenicity, as a pathogenic variant with founder effect in the Ashkenazi Jews population [55].

*ACD* and *TERF2IP* genes are located on chromosome 16q22.1 and 16q23.1, respectively. These genes were identified as risk factors for familial melanoma by a large cohort study carried out on 510 melanoma families [56]. Seven *ACD* and three *TERF2IP* pathogenic/likely pathogenic variants were totally identified supporting the pivotal role of telomere dysregulation in melanoma susceptibility. Moreover, a recent study by Goldstein et al. highlighted the association between germline variants in telomere maintenance genes (*POT1*, *TERF2IP*, *ACD*, and *TERT*) and the spitzoid morphology in familial melanoma [57].

**Figure 2:**
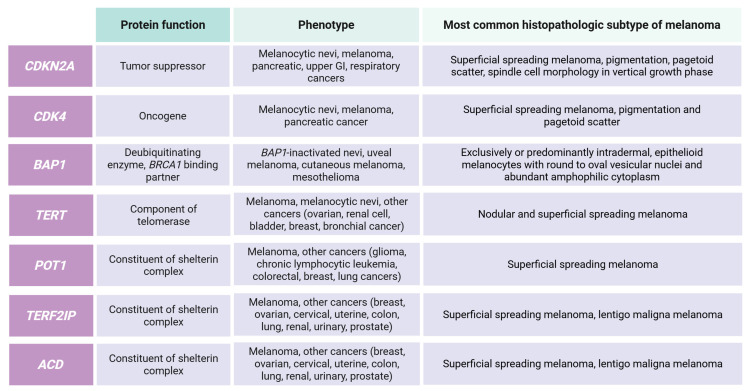
High-risk melanoma susceptibility genes and associated clinical–pathologic features. This figure summarizes key genes implicated in hereditary melanoma predisposition, detailing their protein function, associated phenotypes, and the most common histopathologic subtypes of melanoma.

High-risk genes associated to hereditary melanoma are summarized in Figure 2.

### 3.2. Moderate-Risk Genes

The *MC1R* (Melanocortin 1 Receptor) gene is located on chromosome 16q24.3; the encoded protein, a transmembrane G protein coupled receptor, controls melanogenesis and in particular it plays an important role in normal skin pigmentation [58,59]. In this gene, 3107 variants are known and 42 are classified as pathogenic/likely pathogenic (https://www.ncbi.nlm.nih.gov/clinvar/, accessed on 20 July 2025). Interestingly, some *MC1R* polymorphisms may play a role in familial melanoma risk and it seems to be correlated to the number of inherited variants [7]. According to this finding, a multigene panel testing has been recently carried out on a large Dutch population, showing that *MC1R* risk variants had a strong association with the melanoma respect to the control group [60]. It has to be underlined that *MC1R* variants are common in the general population, and their modest effect size is difficult to isolate due to environmental confounders. Additionally, case-control studies often differ in their inclusion criteria and definitions, which complicates cross-study comparison.

The *MITF* gene (Melanocyte-Inducing Transcription Factor) is located on chromosome 3p13. MITF helps the development and functions of melanocytes. Melanocytes control the production of the pigment melanin, which contributes to hair, eye, and skin color [61]. In total, 90,634 variants were found spanning on the gene and 153 of them show a pathogenic/likely pathogenic interpretation (https://www.ncbi.nlm.nih.gov/clinvar/, accessed on 20 July 2025). To date, only one *MITF* variant, the p.Glu318Lys, has been associated to melanoma susceptibility [62]. The *MITF* p.Glu318Lys mutation not only increases the risk of melanoma, but has also been associated to high nevi count and fast-growing melanomas; interestingly, this mutation can be present also in p16INK4A mutations carriers [63,64]. Potjer et al. detected the *MITF* p.Glu318Lys mutation in 15 melanoma families suggesting a pivotal role of this mutation, at least in the Dutch population [60]. Mutation carriers should be encouraged to strict dermatologic surveillance, periodic self-examination, and renal cell carcinoma surveillance.

Finally, *TERT* variants not occurring in the promoter (see above) can behave as low-risk variants [17].

### 3.3. Low-Risk Genes

Recently, variants in other genes have shown an association with familial melanoma, but the risk conferred by these variants is low and still partly unclear [26,65].

The *MGMT* (O-6-Methylguanine-DNA Methyltransferase) gene is located on chromosome 10q26.3. The protein encoded is involved in the cellular defense against the biological effects of O6-methylguanine (O6-MeG) and O4-methylthymine (O4-MeT). More in detail, it repairs the methylated nucleobase in DNA by transferring the methyl group to a cysteine residue in the enzyme. This gene is considered a susceptibility gene for cutaneous melanoma [66] and a biomarker for glioblastoma and gliosarcoma. Moreover, *MGMT* gene promoter methylation was found in brain metastases of melanoma, lung, breast, and renal carcinoma [67].

Association studies (Genome-wide association studies—GWAS) found many SNPs in a different group of genes all involved in skin pigmentation, in particular, *TPCN2*, *ASIP*, *KITLG*, *SLC24A5*, *SLC45A2*, *TYR*, *IRF4*, *OCA2*, and *TYRP1* genes, as well as the *MC1R* gene already discussed [68,69,70]. These results highlight the importance of pigmentation-related genes in the onset of melanoma and tumorigenesis through genetic–environment interactions. In particular, *TPCN2* is an ion transport gene containing SNPs associated with hair pigmentation that have also been associated to increased melanoma risk, especially in males [71]. The *ASIP* gene inhibits MC1R shifting melanin color to a less protective isoform; this gene has also been associated to high-risk phenotype and melanoma susceptibility [72]. *KITLG* is involved in melanocytes development and maintenance; SNPs in this gene have been associated to altered skin pigmentation and increased risk of melanoma [69]. SLC24A5 transports calcium or potassium ions into the melanosome and plays a role in melanogenesis, while SLC45A2 is a sodium–hydrogen exchanger of melanosomes, regulating tyrosinase activity in human melanocyte; both genes have been proposed as risk factors for melanoma development [73]. TYR catalyzes the conversion of tyrosine to melanin; variants affecting this gene have been related to albinism and increased melanoma risk [64]. *IRF4* is involved in gene expression regulation in response to cytokines and interferon: a SNP in this gene has been associated with increased risk of melanoma [74]. Finally, *OCA2* and *TYRP1* genes are both involved in oculocutaneous albinism: variants affecting these genes have been related to increased susceptibility to familial melanoma [75].

### 3.4. Candidate Genes

A GWAS study, reporting a meta-analysis combining data from 11 previous GWAS and an additional data set, identified 20 significant loci associated to familial melanoma: 13 already known, two previously-reported but not considered significant, and five novels (explaining an additional 2.3% of the familial relative risk) [76]. In particular, one locus includes putative melanocyte and keratinocyte enhancers and the *CYP1B1* gene. *CYP1B1* metabolizes hormones and has been associated to hormone-related cancers, such as prostate and breast cancers [77]. Interestingly, *CYP1B1* is regulated by *ARNT*, a gene present in a melanoma-associated locus, suggesting a common pathogenetic pathway [78]. Another identified locus involves *CDKAL1*, a gene involved in gene expression regulation. The most significant SNP identified in this gene is localized in a predicted melanocyte enhancer that binds *IRF4*: this is intriguing since an *IRF4* SNP (rs12203592) has been associated with nevus count, skin pigmentation, and tanning response [79,80]. Another region involves the *AGR3* gene, a member of the protein disulfide isomerase family, already associated to breast cancer risk and poor survival in ovarian cancer [81,82]. Next, an association was found with a region including *TMEM38B*, *ZNF462,* and *RAD23B* genes, the latter being of particular interest due to its involvement in DNA repair. A locus spanning the *OBFC1* gene and the *SH3PXD2A* promoter was highlighted and since this region is associated with telomere length; this suggests that *OBFC1*, as a member of telomeres maintenance complex, may be a candidate susceptibility gene for familial melanoma [76]. Another locus involves the *CCND1* gene, in which somatic mutations has been already reported as a marker of poor survival in melanoma [76]. Finally, a locus including both *OCA2* and *HERC2* genes was also identified. These genes have been reported to be important determinants of eye color, thus suggesting that they may be potential candidates for susceptibility to familial melanoma [76].

Melanoma is an immunogenic tumor but the relationship between the immune system and the skin cancer is currently under investigation [83,84,85,86]. Indeed, another group of genes, including genes of the immune system, may be implicated in melanoma onset: *IL-10*, *IL-1β*, *TNF-α* (tumor necrosis factor alpha), *HLA* (human leukocyte antigen class II) genes, and *IRF4* (interferon regulatory factor 4) genes [83,84].

Finally, novel genes are being proposed as risk factors for familial melanoma.

A rare non-synonymous variant has been identified in Golgi Membrane Protein 1 (*GOLM1*), rs149739829, in two patients from the same family [87]. The variant co-segregates with the phenotype in this family and was confirmed in independent cases, thus suggesting that *GOLM1* may be a candidate susceptibility gene.

Similarly, an exome sequencing study performed in high-risk patients highlighted two *TP53AIP1* truncating mutations in four patients, suggesting a role in melanoma predisposition [88].

In conclusion, moderate and low susceptibility genes have a weak impact on melanoma onset and families that carry these variants usually have only one or two melanoma cases. Nevertheless, it is possible that a combination of low to moderate inherited variants, also in combination with environmental factors, may contribute to the melanoma carcinogenesis.

### 3.5. Susceptibility Loci

In addition to the well-established and emerging genes associated with an increased risk of familial melanoma, there are also some susceptibility loci that have been identified by GWASs that may harbor other susceptibility genes.

Gillanders et al., by analyzing 49 Australian families with familial melanoma and no mutations in *CDKN2A* and *CDK4* genes, identified a novel melanoma susceptibility locus on Chr1p22, confirmed by the further analysis of other families from other countries [89]. A subsequent GWAS, carried out on 35 Swedish families with no *CDKN2A* mutations, detected a strong association with the region Chr17p12–p11 suggesting that this locus may contain genes involved in melanoma susceptibility, at least in Sweden [90]. A linkage analysis on 34 high-risk families highlighted an association with the region Chr9q21 [91]; interestingly, several genes are present in this region including *GOLM1*, recently described as a candidate gene [87]. Tuominen et al. investigated a putative susceptibility locus in the region Chr3q29 harboring 44 genes that may be novel melanoma-associated genes [92]. Finally, a GWAS carried out in Spanish *CDKN2A*-negative patients identified a novel familial melanoma-associated locus at Chr11 harboring four genes (*DLG2*, *PRSS23*, *FZD4*, and *TMEM135*) with the strongest linkage evidence [93].

A large meta-analysis led by Landi et al. reported 68 independent cutaneous melanoma-associated variants across 54 loci confirming the importance of key functional pathways and highlighting previously unknown cutaneous melanoma etiologic routes [94].

Future studies investigating these regions through next-generation sequencing may allow the identification of novel familial melanoma susceptibility genes.

## 4. Genetic Testing

*CDKN2A* molecular screening is recommended to assess the presence of a melanoma predisposing-syndrome [95]. Although *CDKN2A* and *CDK4* are the most commonly tested genes to date, no unique standard guidelines on which genes to include in familial melanoma genetic testing are currently available. According to NCCN Guidelines (Version 2.2025, accessed on July 2025), genetic predisposition is defined by the presence of germline mutations or polymorphisms predisposing to melanoma (e.g., *CDKN2A*, *CDK4, MC1R*, *BAP1* [especially for uveal melanoma], *TERT*, *MITF*, and *PTEN*) and other cancer predisposition genes with increased melanoma risk (e.g., *CHEK2*, *BRCA1/2*, *BLM*, and *ATM*).

In addition, the availability of next-generation sequencing (NGS)-based approaches, able to comprehensively analyze more candidate genes (from a few to the whole exome) in several patients simultaneously, has enhanced the diffusion of molecular analyses in a diagnostic context [96,97,98,99]. Indeed, this approach enables, with an acceptable cost and in a relatively short time, gaining multiple information regarding the presence of causative mutations, risk factors, drug response-related variants, and phenotype-modifiers variants, thus stratifying patients and identifying the at-risk subjects within their families. In addition, by analyzing a group of candidate genes in large cohorts, it is also possible to estimate the contribution of a specific gene to a disease of interest or discover novel associations. As a consequence, multi-gene panels analysis is becoming the best option for the molecular diagnosis of several inherited and genetically heterogeneous diseases, like hereditary cancers [11,12,100,101,102].

The main advantages of a molecular diagnosis for familial melanoma are the possibility to make patients aware about their cancer risk and contributing risk factors, and plan more frequent surveillance in order to make early diagnosis and modulate the melanoma risk in other family members [103].

Considering all the above, gene panels testing is becoming a common practice also in patients affected by melanoma in order to assess an inherited predisposition. However, considering the low frequency of melanoma-related germline mutations, their variable penetrance, and the absence of standardized guidelines for the clinical management of the mutations’ carriers, the use of these tests as a diagnostic tool is currently under debate. In addition, since the prevalence of predisposing mutations is different in different geographic areas, this makes it difficult to establish a common panel for all countries. Finally, expanding the number of genes included in testing inevitably increases the likelihood of identifying variants of uncertain significance (VUSs). These findings may lead to inconclusive results, complicate risk communication, and create uncertainty for both clinicians and patients. Indeed, the detection of VUSs represents a known limitation of broad genetic testing approaches. While such variants may be reclassified over time, their initial ambiguity poses challenges in clinical decision-making and genetic counseling. It is therefore essential to balance diagnostic yield with the risk of generating uncertain results.

Thus, it is important to underline that genetic counselling should always be offered to patients in the presence of a suspicion of a hereditary cancer syndrome, including familial melanoma [23,95]. Indeed, patients should be informed about the significance of the test, the probability of inconclusive results, and the possible personal and familial implications. Aiming to reduce uninformative tests, Leachman et al. proposed a diagnostic algorithm for the molecular analysis of hereditary susceptibility to melanoma based on the separation between “melanoma dominant” (the melanoma is the most frequent cancer in the family) and “melanoma subordinate” (the melanoma is secondary respect to other predominant cancers in the family) cases [23]. According to this classification, they suggested different gene panels to be tested based on the penetrance of the genes for the cancer found as predominant in the patient’s family.

**Figure 3:**
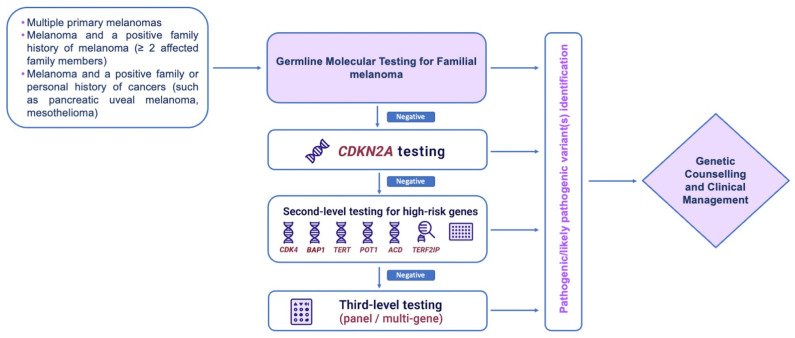
Diagnostic algorithm for familial melanoma. In the presence of clinical suspicion for familial melanoma, based on a patient’s personal and family history, molecular testing to verify the presence of a germline predisposing variant should be considered. Given the frequency and penetrance of specific gene mutations—and the availability of clinical guidelines for mutation carriers—a stepwise approach is recommended. This includes first-level testing of *CDKN2A*, followed by the analysis of the other high-risk genes. In patients with strong clinical indications but negative results at earlier steps, broader testing, such as enlarged multigene panels or whole-exome sequencing, may be appropriate. This flowchart aims to balance comprehensive testing with the need to avoid unnecessary analyses and reduce the risk of uncertain results.

Potjer et al. used a custom panel, including 30 genes, to analyze 488 melanoma cases from *CDKN2A* and *CDK4*-negative families [60]. They were able to identify 18 pathogenic/likely pathogenic variants (3 in *BAP1* and 15 in *MITF*) obtaining a 4% increase of the diagnostic yield. Their results not only support the use of a multi-genes panel test in familial melanoma, but also suggests a pivotal role of *BAP1* and *MITF* genes. More recently, Bruno et al. successfully used multi genes panel testing for familial melanoma evaluation and, by analyzing 1044 family members and 940 cutaneous melanoma index cases, found that *CDKN2A* pathogenic variants’ detection rate was 5.53% (52 cases), while adding the variants identified in the other genes, the panel diagnostic sensitivity increases up to 9.47% (89 cases) [104]. Based on all the reviewed studies and current NCCN guidelines, a possible diagnostic algorithm for familial melanoma is reported in Figure 3.

## 5. Genetic Counseling and Mutations Carriers’ Management

The burden of melanoma-risk genes is becoming even more clear with the emergence of other predisposing genes and the diffusion of extended genomic analyses that allow multiple genetic loci to be analyzed simultaneously.

Thus, pre-test genetic counseling should be mandatory to explain the significance of the test proposed, the possible results, and their significance for the proband and for the family. Similarly, the post-test genetic counseling has to be carried out to discuss the results and their repercussions in the familial context. Indeed, the combination of the appropriate genetic test in properly selected melanoma patients and the personalized recommendation for follow-up based on the test results has shown to both improve early detection and reduce mortality [23]. This means that the selection criteria to access genetic testing are crucial in order to identify those patients that are more likely to carry a germline predisposing mutation. However, established guidelines for melanoma-predisposing genes testing are not yet available.

About fifteen years ago, a pre-test probabilities estimation was proposed for the identification, within the melanoma patients, of proper candidates for the molecular analysis [15]. This study suggested using as criteria the number of primary melanomas in the proband, the number of melanomas in blood relatives, and the numbers of pancreatic cancers in the proband or blood relatives, normalizing these criteria for the geographic area. This is the so-called “rule of twos or threes” for melanoma molecular testing. This rule has been recently updated to include the other cancers that may co-occur in the families; interestingly, based on this a priori probability, authors suggest different gene panels to be assessed in different patients [23].

In addition, even if today genomic screening methods allow comprehensive genomic analyses to be carried out, it is important to underline that their results may be inconclusive. Indeed, a number of VUSs may be identified. Lastly, for more known pathogenetic mutations, established guidelines for mutations carriers’ clinical management and follow-up may be lacking. In an attempt to minimize this uncertainty, Leachman et al. suggest reporting only genes and variants with established clinical significance [23].

In the presence of positive family history or of an identified predisposing mutation, melanoma patients should receive specific recommendations for follow-up and therapy. First of all, patients should be advised on the importance of melanoma prevention and early detection: photoprotection has to be encouraged, as well as self-skin examination. In addition, the frequency of dermatological visit must be planned based on the patient’s history and skin features.

Dermoscopy, in particular, is an important instrument for the monitoring of these patients, also considering the variability of the phenotype [16,105]. Indeed, most studies suggested that an interval of 6 months between subsequent dermoscopies should be adequate [106,107], while Haenssle et al. suggested to reduce it to 3 months [108]; to date, there are still inconclusive data supporting that this reduction may ameliorate patients’ outcome. Other advanced techniques, like total body photography and sequential digital dermoscopy imaging, may be also useful for patients at extreme risk of melanoma [109].

A study carried out on 311 high-risk patients evaluated at 6-month intervals, by using total body photography and sequential digital dermoscopy imaging, showed an improvement in post-baseline melanoma detection [110]. In addition, it has been reported that the use of total body photography and sequential digital dermoscopy imaging allows diagnosis of thinner melanomas in respect of those identified by physical examination [111]. Also in this case, the frequency of follow-up is still under debate. A cost-analysis study has demonstrated that the cost for the surveillance of the high-risk subjects, even if high, are cheaper than the cost to treat a melanoma [112].

Considering that familial melanoma is associated often to an early age of onset, the dermatological surveillance should begin from adolescence.

Annual screening for other cancers should be also evaluated depending on family history and on the spectrum of cancer related to the mutated gene.

Finally, in the presence of a predisposing mutation within a melanoma family, the blood relatives of the proband should be admitted to genetic counseling in order to receive adequate information regarding their own risk, the most appropriate surveillance strategies and evaluate the possibility of undergoing genetic analysis to exclude or confirm the inheritance of the familial mutation. It is important to underline that, as for other cancers, inherited mutations confer a risk and do not allow a melanoma diagnosis to be made or evaluate if and when a melanoma may develop. Over time, some risk scores have been developed in an attempt to evaluate personal melanoma risk. In particular, Davies et al. developed a melanoma risk algorithm based on the evaluation of seven phenotypic features (hair color, skin type, family history, freckling, nevus count, number of large nevi, and history of sunburn) available as a web tool for self-assessment [113]. Subsequently, Cho et al. tested the use of a genetic risk score based on the use of 21 SNPs reported as significantly related to melanoma risk; however, this study highlighted that, despite the associated risk, genetic factors seem to have a limited impact on melanoma development with respect to other environmental factors [114]. To deeply investigate the contribution of genetic factors to melanoma risk, a study derived a polygenic risk score from the analysis of two independent cohorts: interestingly, they found that genetic factors evaluation may identify at-risk subjects in absence of typical phenotypic features [115]. Finally, Gu et al. reported a polygenic risk score combining 204 SNPs that, in combination with skin pigmentation features and the number of nevi, showed its potential in the identification of the high-risk subjects who could benefit from increased surveillance [116]. Further studies and prospective analyses on different populations are required to establish the use of these scores and their utility in familial melanoma management.

Currently, specific dermatological surveillance recommendations are available only for carriers of mutations in a few high-risk genes, primarily *CDKN2A* (see Table 2). These protocols typically include full-body skin examinations every 3–12 months, beginning in early adulthood. For the other familial melanoma susceptibility genes, evidence remains insufficient to support standardized surveillance guidelines [14].

## 6. Role of Germline Variants in Prognosis and Therapy

Germline-inherited DNA variants, in addition to increasing the risk for developing melanoma, may also play a role in patients’ prognosis and/or survival. The identification of germline variants able to affect melanoma survival enables an increase in the knowledge on the molecular bases of melanoma. In particular, specific key pathways may be identified, supporting the development of novel therapies for the treatment of melanoma cases with a poor prognosis. To this attempt, the GenoMel consortium (https://genomel.org/, accessed on 20 July 2025) was launched and is investigating the role of inherited DNA variants on melanoma survival.

In this context, a BioGenoMel collaborative study aimed at verifying the hypothesis that inherited *MC1R* variants may impact melanoma survival expectation [117]. By analyzing 10 melanoma cohorts from different countries, they suggested a survival benefit for melanoma patients carrying inherited specific *MIC1R* variants [118]. Subsequently, the association between *MIC1R* variants and survival rate has been further investigated by an international, large population-based project within the Genes, Environment, and Melanoma study [117]. By analyzing the *MIC1R* genotype in 2200 patients with a primary melanoma, this study suggested that melanoma survival may be improved in the carriers of two inherited variants in the *MIC1R* gene [118]. Finally, a study by Lira et al. investigated the association between *MC1R* variants, melanoma survival, and sex [119]. Interestingly, they found that inherited *MC1R* variants correlated with an improved overall survival rate only in women, suggesting that sex-dependent features may influence the role of specific genes involved in melanoma prognosis [119].

In addition to *MIC1R*, germline variants in other genes have been identified as predictive of melanoma patients’ outcome. In particular, the same population-based study carried out in the context of the Genes, Environment, and Melanoma study, mentioned above [118], aimed to evaluate the possible role on melanoma survival of polymorphisms located in the *ASIP* (Agouti signaling protein) gene. Indeed, *ASIP* protein is the antagonist of MC1R and GWASs have highlighted an association between *ASIP* polymorphisms and the risk of melanoma [68,120]. In this study, the *ASIP* risk haplotype TG was found to significatively affect melanoma survival with respect to the most common GG haplotype [118].

Davies at al. reported an association between a rare, inherited variant in the *PARP1* (polyadenosine diphosphate-ribose polymerase 1) gene and melanoma survival. This study showed that the investigated *PARP1* SNP was associated with increased survival of melanoma patients and that the reduced expression of *PARP1*, affecting both melanoma growth and angiogenesis, may positively impact on melanoma progression [121].

SNPs in the *VDR* (Vitamin D Receptor) gene have been reported to affect the survival rate of several cancers [122,123]. Thus, the Genes, Environment, and Melanoma study also aimed to assess their role in melanoma-affected patients [124]. By analyzing 3566 primary melanoma cases, this study found that *VDR* SNPs may affect melanoma survival even if tumor aggressiveness was not modified [124].

The *IRF4* (Interferon regulatory factor 4) gene encodes for a transcription factor within the interferon regulatory family and is expressed in the melanocytic lesions [125]. An *IRF4* SNP has been also associated to an increased risk of melanoma [80,126]. Poltrony et al. (2017) aimed to verify the role of this *IRF4* SNP in melanoma patients’ outcome [127]. By analyzing two independent cohorts, this study found that the *IRF4* SNP was associated with a worse melanoma outcome [127].

Finally, some studies have also investigated the different survival in familial melanoma patients according to their *CDKN2A* mutation status [128,129,130,131]. In particular, Florell et al. aimed to demonstrate different prognosis and survival rates in familial melanoma with respect to the sporadic cases [128]. They carried out a large population-based study that did not highlight any different biological behavior between the two studied groups. Instead, De Giorgi et al. reported different clinical and prognostic features in melanoma patients carrying a *CDKN2A* mutation with respect to the non-carriers, the latter showing the worst prognosis [129]. On the contrary, Helgadottir et al., comparing the survival in germline *CDKN2A* mutated and nonmutated melanoma cases, found the *CDKN2A* mutated cases had statistically significantly worse survival than melanoma cases with no *CDKN2A* mutations [130]. Finally, a subsequent study, confirming the previous report from De Giorgi et al. (2015), reported no difference on the overall survival between *CDKN2A* mutated and nonmutated melanoma cases, at least in the Italian population [131].

In addition to the above-mentioned role on melanoma prognosis and survival, germline-inherited variants may also be involved in patients’ response to specific therapies.

Indeed, as for other cancers, melanoma therapies include different approaches, the choice being based on several factors, including the patient’s health, tumor stage, and location. In addition to the classic protocols (surgical resection, chemotherapy, photodynamic therapy, and biochemotherapy), targeted therapy, and immunotherapy were recently approved for metastatic melanoma [132,133,134]. Targeted therapy refers to a group of drugs able to inhibit specific pathways by acting on specific somatic mutations, such as the MAPK pathway in patients that carry the V600E/K in *BRAF* gene [135].

Immunotherapy, instead, acts through the inhibition of different cellular checkpoints that directly activate the patient’s immune system [136]. More details can be found in specific papers focusing on these aspects [132,137,138,139,140,141,142,143,144,145,146].

All these therapeutic approaches are targeted to somatic melanoma-related hallmarks and little is known regarding their efficacy in melanoma patients carrying germline predisposing variants.

In this context, Helgadottir et al. evaluated the efficacy of immunotherapy agents in *CDKN2A* germline mutation carriers affected by metastatic melanoma. Interestingly, these patients showed a better outcome and a higher efficacy of immunotherapy with respect to nonmutated patients. This finding was probably due to high neoantigens production resulting in high sensitivity to immunotherapy, as a consequence of the high mutation load in the presence of a germline predisposition [147]. Similarly, Guida et al. evaluated the effects of *MC1R* variants on *BRAF*-targeted therapy. They found that the presence of *MC1R* variants significantly correlate with a worse outcome considered as both overall response rate and progression free survival [148].

Finally, a recent paper by Chat et al. showed that germline autoimmunity risk factors are able to influence the response to melanoma immune-checkpoint inhibition, suggesting that systematic testing of autoimmune risk loci could be useful to identify personalized biomarkers predictive of immunotherapy response [149].

## 7. Discussion and Conclusions

Autosomal dominant hereditary mutations have been identified as genetic predisposing factors for melanoma [26,150]. To date, up to 15% of melanoma cases are considered familial and may be related to germline predisposing mutations, *CDKN2A* being the most common and highly penetrant gene [151]. Nevertheless, single gene testing for the evaluation of familial melanoma risk is under debate. Indeed, considering that nonspecific therapies targeting the mutations are available (if not for somatic mutations), the clinical relevance of these tests is not clearly established. On the other hand, the possibility of using multi-gene panels at reasonable costs reduces the interest for single-gene testing.

While it is obvious that the greater the number of genes analyzed, the higher the risk for inconclusive results or difficult-to-interpret variants [152], it is also true that the more the genes panel analysis will be extended, the more our knowledge about the molecular landscape of hereditary susceptibility to melanoma will increase.

In addition, it is noteworthy that most of our knowledge about germline mutations predisposing to familial melanoma is focused on mutations occurring as a consequence of single nucleotide substitutions or small insertions/deletions, while little is known regarding the role of copy number variants (CNVs). As for other diseases, the frequency of CNVs and their role in melanoma progression may be underestimated [153]. Yang et al. identified in all the affected members of a melanoma family a duplicated region on chromosome 4, containing genes encoding CXC chemokines, and suggested their possible involvement in melanoma susceptibility [154]. Recently, Rocca et al. reported that germline CNVs in the gene encoding for the E2F1 transcription factors, that is known to play a role in cell cycle regulation and apoptosis, may be a predisposing factor to melanoma [155]. The use of specific bioinformatic software for NGS data, to be confirmed by an independent quantitative method (quantitative PCR, MLPA or a-CGH), has already shown its reliability and may further improve our comprehension regarding the molecular bases of familial melanoma. Moreover, it is becoming evident that genomic variants may not be the sole molecular mechanism underlying genetic predisposition to familial melanoma but epigenetic factors may also play a role. In particular, *MGMT* promoter methylation has received increasing attention. Hypermethylation of the *MGMT* gene may contribute to impaired DNA repair capacity, increased mutational burden, and a potentially altered response to alkylating agents. While not yet integrated into routine clinical management, *MGMT* methylation status may represent a promising biomarker deserving further investigation. This growing body of knowledge will provide valuable insights not only for a better identification of the molecular bases of familial melanomas, but also for at-risk subjects’ ascertainment, and for the development of novel targeted therapies, as already done for other hereditary cancer syndromes [156,157].

In conclusion, while *CDKN2A* remains the most established high-penetrance gene for familial melanoma, recent findings on other genes have broadened the genetic landscape and complexity of this condition. However, current knowledge on familial melanoma susceptibility is largely centered around a few high-penetrance genes, such as *CDKN2A*, *CDK4*, *BAP1,* and *POT1*, for which clinical management guidelines are available.

The growing use of multigene panels, including moderate-, low-risk, and emerging genes, has improved mutations detection but raises challenges, such as the careful interpretation of VUSs and awareness of the limitations associated with uncertain findings. As highlighted in this review, the inclusion of CNVs evaluation and the refinement of bioinformatic pipelines are crucial steps toward a more comprehensive assessment of genetic risk. Moreover, surveillance protocols are currently limited to a few genes and should be expanded as new evidence emerges. Improving genetic counseling, standardizing testing strategies, and integrating CNVs analysis will be crucial steps toward optimizing patient care and developing tailored prevention strategies. The diagnostic algorithm we propose (Figure 3) integrates current evidence and may serve as a guide for clinicians in tailoring genetic testing and surveillance strategies in individuals and families at increased risk of melanoma. Future research should focus on better characterizing moderate-penetrance genes, clarifying the role of CNVs, and refining genotype-based clinical recommendations.

## Figures and Tables

**Table 1:** Genes related to hereditary melanoma reported according to their chromosomal location and penetrance.

Gene	Name	Chr * Localization	Penetrance
*CDKN2A*	Cyclin-Dependent Kinase Inhibitor 2A	9p21.3	High
*CDK4*	Cyclin-Dependent Kinase 4	12q14.1	High
*BAP1*	BRCA1-Associated Protein 1	3p21.1	High
*TERT*	Telomerase Reverse Transcriptase	5p15.33	High
*POT1*	Protection of Telomeres 1	7q31.33	High
*ACD*	ACD Shelterin Complex Subunit and Telomerase Recruitment Factor	16q22.1	High
*TERF2IP*	TERF2-Interacting Protein	16q23.1	High
*MC1R*	Melanocortin 1 Receptor	16q24.3	Moderate
*MITF*	Melanocyte-Inducing Transcription Factor	3p13	Moderate
*MGMT*	O-6-Methylguanine-DNA Methyltransferase	10q26.3	Low
*TPCN2*	Two Pore Segment Channel 2	11q13.3	Low
*ASIP*	Agouti Signaling Protein	20q11.22	Low
*KITLG*	KIT Ligand	12q21.32	Low
*SLC24A5*	Solute Carrier Family 24 Member 5	15q21.1	Low
*SLC45A2*	Solute Carrier Family 45 Member 2	5p13.2	Low
*TYR*	Tyrosinase	11q14.3	Low
*IRF4*	Interferon Regulatory Factor 4	6p25.3	Low
*OCA2*	OCA2 Melanosomal Transmembrane Protein	15q12-q13.1	Low
*TYRP1*	Tyrosinase-Related Protein 1	9p23	Low
*CYP1B1*	Cytochrome P450 family 1 subfamily B member 1	2p22.2	Low
*ARNT*	Aryl Hydrocarbon Receptor Nuclear Translocator	1q21.3	Low
*CDKAL1*	CDKAL1 threonylcarbamoyladenosine tRNA methylthiotransferase	6p22.3	Low
*AGR3*	Anterior Gradient 3, protein disulfide isomerase family member	7p21.1	Low
*TMEM38B*	Transmembrane protein 38B	9q31.2	Low
*ZNF462*	Zinc Finger protein 462	9q31.2	Low
*RAD23B*	RAD23 nucleotide excision repair protein B	9q31.2	Low
*OBFC1 (STN1)*	STN1 subunit of CST complex	10q24.33	Low
*SH3PXD2A*	SH3 and PX domains 2A	10q24.33	Low
*CCND1*	Cyclin D1	11q13.3	Low
*HERC2*	HECT and RLD domain containing E3 ubiquitin protein ligase 2	15q13.1	Low
*GOLM1*	Golgi membrane protein 1	9q21.33	Low
*TP53AIP1*	Tumor Protein p53 regulated Apoptosis Inducing Protein 1	11q24.3	Low
*IL-10*	Interleukin 10	1q32.1	Low
*IL-1β*	Interleukin 1 Beta	2q14.1	Low
*TNF-α*	Tumor Necrosis Factor	6p21.33	Low

* Chr, chromosome.

**Table 2:** Clinical recommendation for dermatological surveillance in individuals carrying a pathogenic/likely pathogenic variant.

Gene	Cancer Risk Management
*CDKN2A*	- Comprehensive skin examination by a dermatologist with total body photography and dermoscopy every 6 months. - Pancreatic cancer screening from age 40 with annual contrast-enhanced MRI/magnetic resonance cholangiopancreatography (MRCP) and/or endoscopic ultrasound (EUS). - Multidisciplinary surveillance may include annual full-body and brain MRI depending on individuals’ presentation and/or family history.
*CDK4*	- Dermatological examination with dermoscopy every 3, 6, or 12 months depending on individual risk. - No established guidelines for extra-cutaneous cancer surveillance.
*BAP1*	- Full skin examination with dermoscopy every 6 months starting at age 18. - Annual ophthalmologic examination starting at age 16. - Chest ultrasound (US) or MRI, abdomen, and urinary tract US every 2 years from age 30.
*POT1*	- Annual dermatological examination with dermoscopy. - Annual physical examination and complete blood count. - Neck US every 2 years and full-body MRI annually.

## Data Availability

No new data were created or analyzed in this study.

## Abbreviations

The following abbreviations are used in this manuscript:

*ACD*	ACD Shelterin Complex Subunit and Telomerase Recruitment Factor
*AGR3*	Anterior Gradient 3
*ARNT*	Aryl Hydrocarbon Receptor Nuclear Translocator
*ASIP*	Agouti Signaling Protein
*BAP1*	BRCA1-Associated Protein 1
*BRAF*	B-Raf Proto-Oncogene, Serine/Threonine Kinase
*CCND1*	Cyclin D1
*CDK4*	Cyclin-Dependent Kinase 4
*CDKAL1*	CDK5 Regulatory Subunit-Associated Protein 1-Like 1
CDKN2A	Cyclin-Dependent Kinase Inhibitor 2A
CNV	Copy Number Variant
DC	Cyclin D–CDK4 Complex
FAMMM	Familial Atypical Multiple Mole Melanoma
GWAS	Genome-Wide Association Study
*HERC2*	HECT and RLD Domain Containing E3 Ubiquitin Protein Ligase 2
IL	Interleukin
*IRF4*	Interferon Regulatory Factor 4
*MC1R*	Melanocortin-1 Receptor
*MGMT*	O6-Methylguanine-DNA Methyltransferase
*MITF*	Melanocyte Inducing Transcription Factor
NGS	Next Generation Sequencing
*OCA2*	Oculocutaneous Albinism II
*OBFC1*	Oligosaccharide-Binding Fold Containing 1
PARP1	Poly (ADP-Ribose) Polymerase 1
*POT1*	Protection of Telomeres 1
*PTEN*	Phosphatase and Tensin Homolog
*RAD23B*	RAD23 Homolog B
*SH3PXD2A*	SH3 and PX Domains 2A
*SLC24A5*	Solute Carrier Family 24 Member 5
*SLC45A2*	Solute Carrier Family 45 Member 2
SNP	Single Nucleotide Polymorphism
*TERT*	Telomerase Reverse Transcriptase
*TERF2IP*	TERF2 Interacting Protein
*TPCN2*	Two-Pore Segment Channel 2
*TP53*	Tumor Protein P53
*TP53AIP1*	Tumor Protein P53 Regulated Apoptosis Inducing Protein 1
*TYR*	Tyrosinase
*TYRP1*	Tyrosinase Related Protein 1
VDR	Vitamin D Receptor
VUS	Variant of Uncertain Significance
ZNF462	Zinc Finger Protein 462
